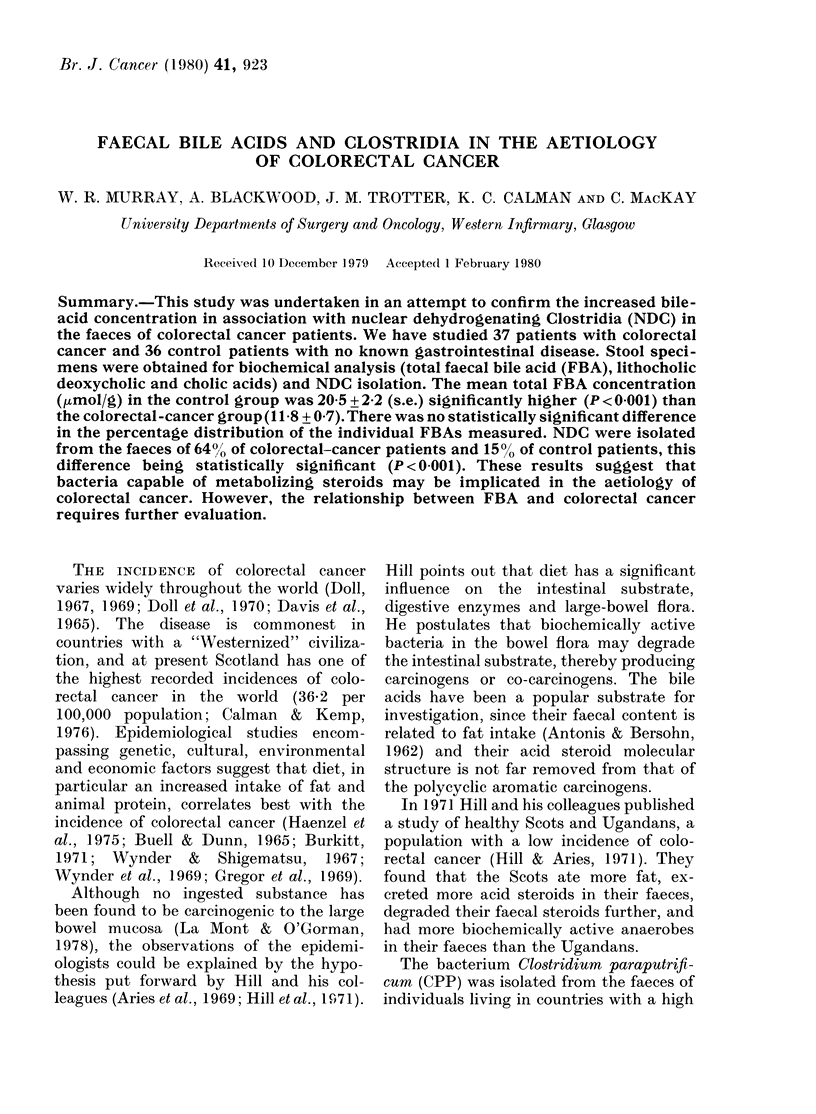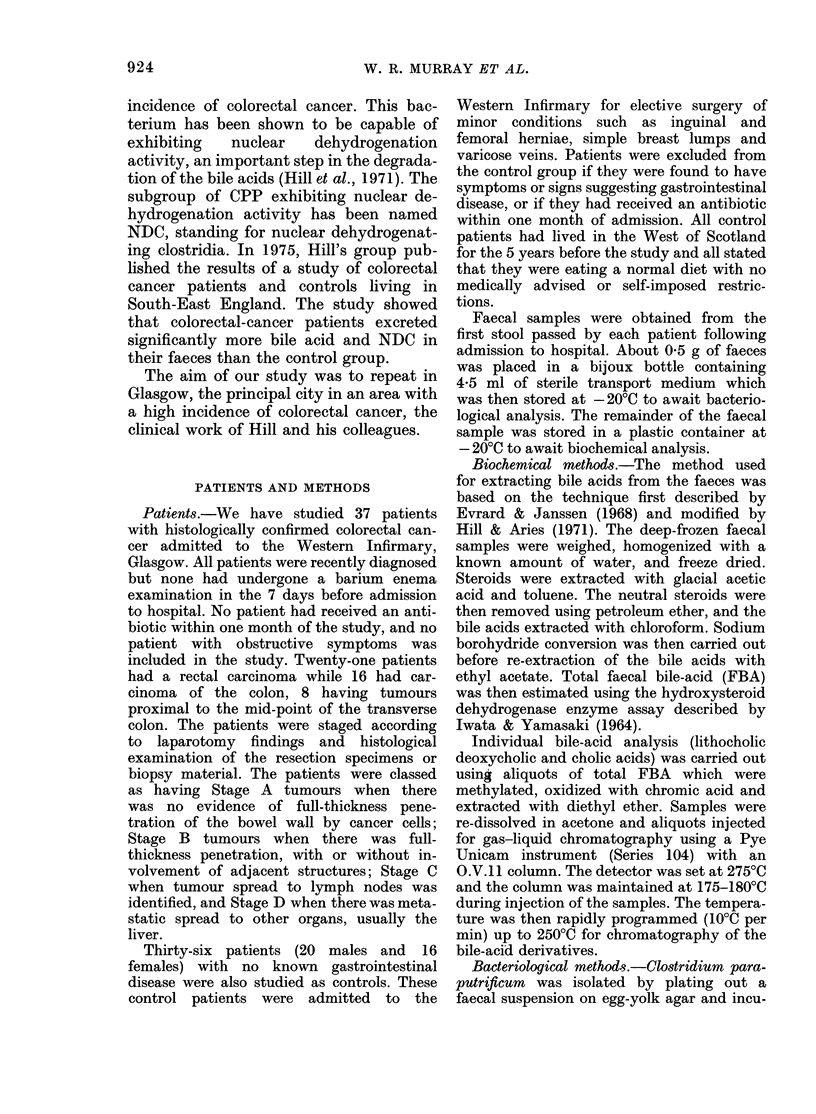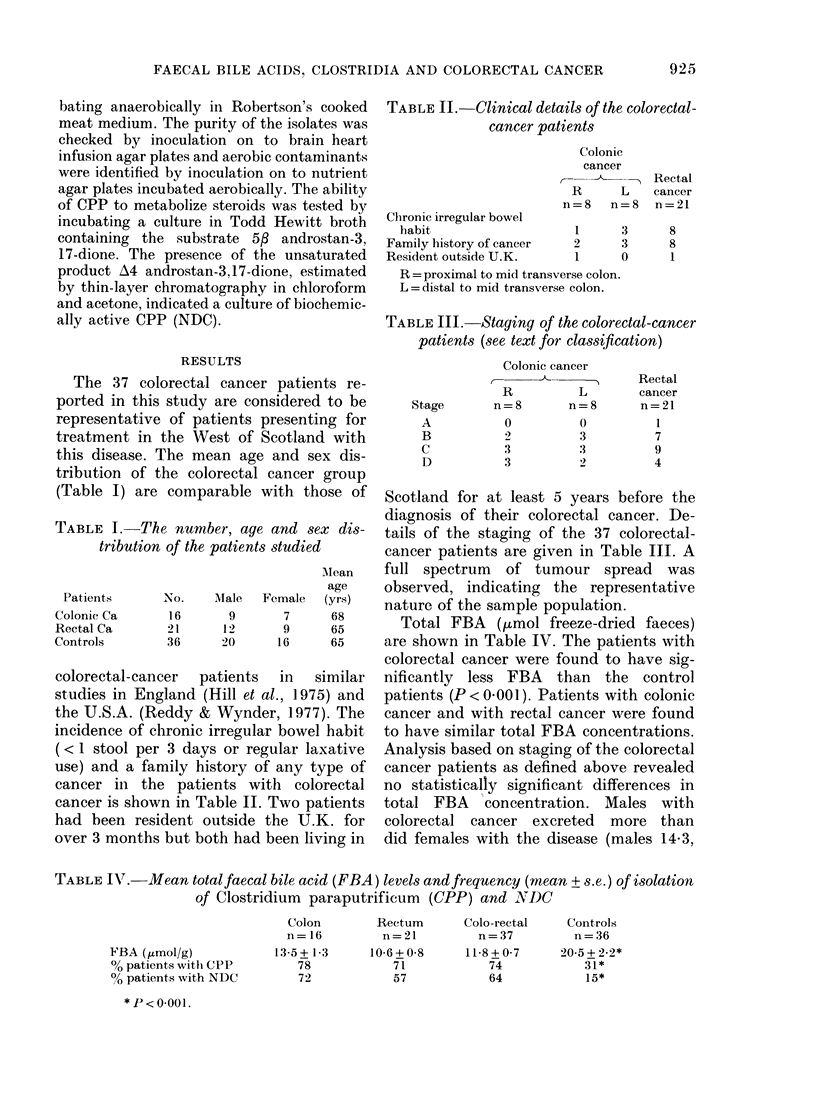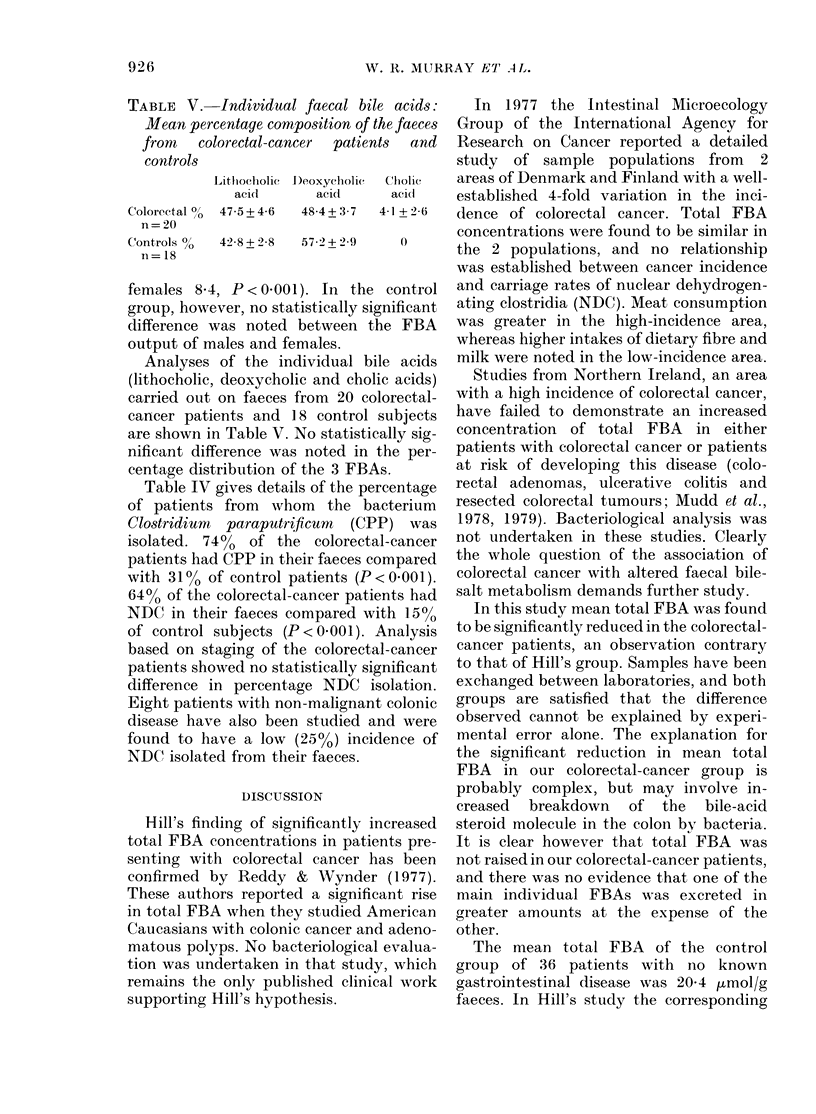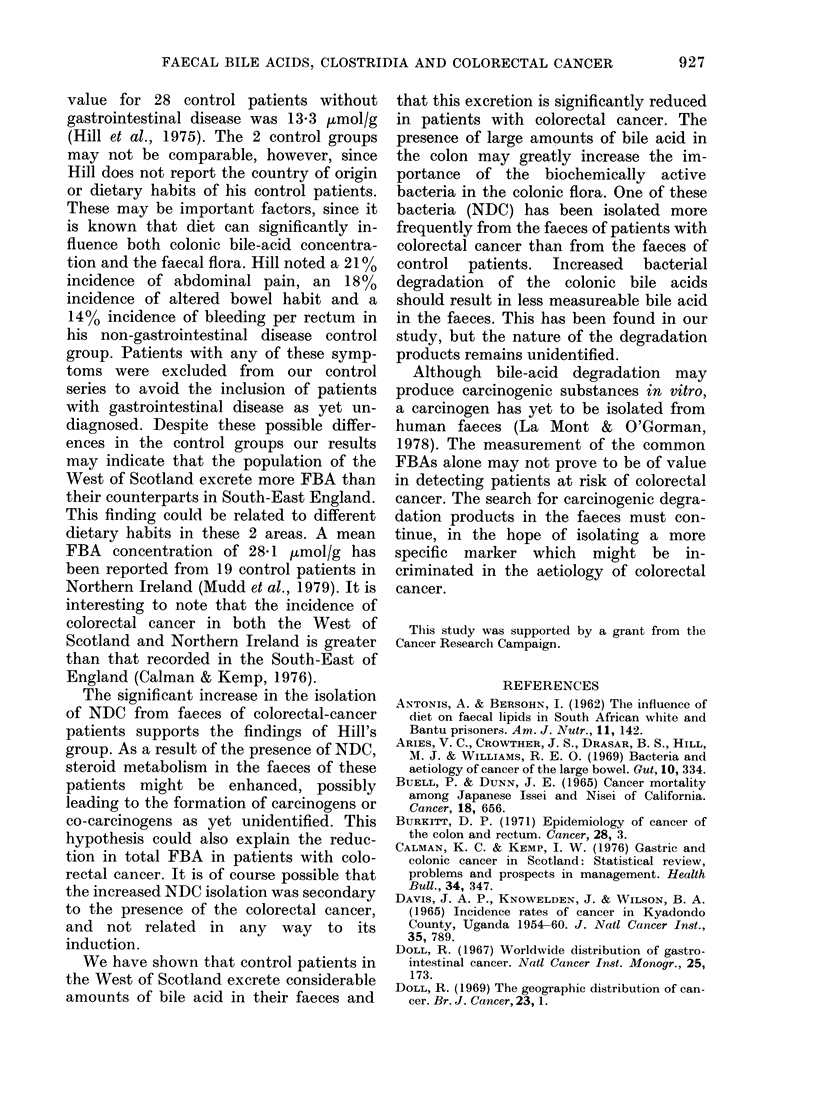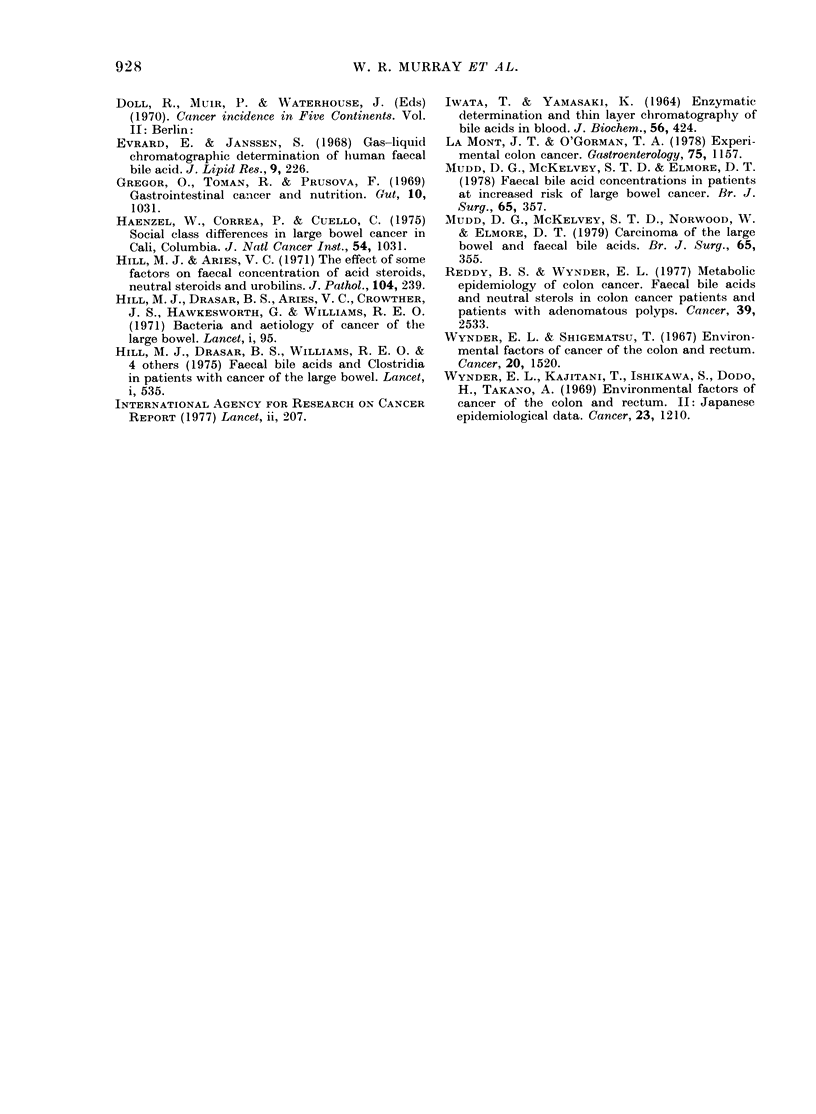# Faecal bile acids and clostridia in the aetiology of colorectal cancer.

**DOI:** 10.1038/bjc.1980.170

**Published:** 1980-06

**Authors:** W. R. Murray, A. Backwood, J. M. Trotter, K. C. Calman, C. MacKay

## Abstract

This study was undertaken in an attempt to confirm the increased bile-acid concentration in association with nuclear dehydrogenating Clostridia (NDC) in the faeces of colorectal cancer patients. We have studied 37 patients with colorectal cancer and 36 control patients with no known gastrointestinal disease. Stool specimens were obtained for biochemical analysis (total faecal bile acid (FBA), lithocholic deoxycholic and cholic acids) and NDC isolation. The mean total FBA concentration (mumol/g) in the control group was 20.5 +/- 2.2 (s.e.) significantly higher (P < 0.001) than the colorectal-cancer group (11.8 +/- 0.7). There was no statistically significant difference in the percentage distribution of the individual FBAs measured. NDC were isolated from the faeces of 64% of colorectal-cancer patients and 15% of control patients, this difference being statistically significant (P < 0.001). These results suggest that bacteria capable of metabolizing steroids may be implicated in the aetiology of colorectal cancer. However, the relationship between FBA and colorectal cancer requires further evaluation.


*Br. J. Cancer* (1980) **41**, 923

### FAECAL BILE ACIDS AND CLOSTRIDIA IN THE AETIOLOGY OF COLORECTAL CANCER

W. R. MURRAY, A. BLACKWOOD, J. M. TROTTER, K. C. CALMAN AND C. MACKAY

*University Departments of Surgery and Oncology, Western Infirmary, Glasgow*

Received 10 December 1979 Accepted 1 February 1980

**Summary.—This study was undertaken in an attempt to confirm the increased bile-acid concentration in association with nuclear dehydrogenating Clostridia (NDC) in the faeces of colorectal cancer patients. We have studied 37 patients with colorectal cancer and 36 control patients with no known gastrointestinal disease. Stool specimens were obtained for biochemical analysis (total faecal bile acid (FBA), lithocholic deoxycholic and cholic acids) and NDC isolation. The mean total FBA concentration (μmol/g) in the control group was 20·5 ± 2·2 (s.e.) significantly higher ($P < 0{\cdot}001$) than the colorectal-cancer group (11·8 ± 0·7). There was no statistically significant difference in the percentage distribution of the individual FBAs measured. NDC were isolated from the faeces of 64% of colorectal-cancer patients and 15% of control patients, this difference being statistically significant ($P < 0{\cdot}001$). These results suggest that bacteria capable of metabolizing steroids may be implicated in the aetiology of colorectal cancer. However, the relationship between FBA and colorectal cancer requires further evaluation.**

THE INCIDENCE of colorectal cancer varies widely throughout the world (Doll, 1967, 1969; Doll *et al.*, 1970; Davis *et al.*, 1965). The disease is commonest in countries with a "Westernized" civilization, and at present Scotland has one of the highest recorded incidences of colorectal cancer in the world (36·2 per 100,000 population; Calman & Kemp, 1976). Epidemiological studies encompassing genetic, cultural, environmental and economic factors suggest that diet, in particular an increased intake of fat and animal protein, correlates best with the incidence of colorectal cancer (Haenzel *et al.*, 1975; Buell & Dunn, 1965; Burkitt, 1971; Wynder & Shigematsu, 1967; Wynder *et al.*, 1969; Gregor *et al.*, 1969).

Although no ingested substance has been found to be carcinogenic to the large bowel mucosa (La Mont & O'Gorman, 1978), the observations of the epidemiologists could be explained by the hypothesis put forward by Hill and his colleagues (Aries *et al.*, 1969; Hill *et al.*, 1971). Hill points out that diet has a significant influence on the intestinal substrate, digestive enzymes and large-bowel flora. He postulates that biochemically active bacteria in the bowel flora may degrade the intestinal substrate, thereby producing carcinogens or co-carcinogens. The bile acids have been a popular substrate for investigation, since their faecal content is related to fat intake (Antonis & Bersohn, 1962) and their acid steroid molecular structure is not far removed from that of the polycyclic aromatic carcinogens.

In 1971 Hill and his colleagues published a study of healthy Scots and Ugandans, a population with a low incidence of colorectal cancer (Hill & Aries, 1971). They found that the Scots ate more fat, excreted more acid steroids in their faeces, degraded their faecal steroids further, and had more biochemically active anaerobes in their faeces than the Ugandans.

The bacterium *Clostridium paraputrificum* (CPP) was isolated from the faeces of individuals living in countries with a high incidence of colorectal cancer. This bacterium has been shown to be capable of exhibiting nuclear dehydrogenation activity, an important step in the degradation of the bile acids (Hill *et al.*, 1971). The subgroup of CPP exhibiting nuclear dehydrogenation activity has been named NDC, standing for nuclear dehydrogenating clostridia. In 1975, Hill's group published the results of a study of colorectal cancer patients and controls living in South-East England. The study showed that colorectal-cancer patients excreted significantly more bile acid and NDC in their faeces than the control group.

The aim of our study was to repeat in Glasgow, the principal city in an area with a high incidence of colorectal cancer, the clinical work of Hill and his colleagues.

### PATIENTS AND METHODS

*Patients.*—We have studied 37 patients with histologically confirmed colorectal cancer admitted to the Western Infirmary, Glasgow. All patients were recently diagnosed but none had undergone a barium enema examination in the 7 days before admission to hospital. No patient had received an antibiotic within one month of the study, and no patient with obstructive symptoms was included in the study. Twenty-one patients had a rectal carcinoma while 16 had carcinoma of the colon, 8 having tumours proximal to the mid-point of the transverse colon. The patients were staged according to laparotomy findings and histological examination of the resection specimens or biopsy material. The patients were classed as having Stage A tumours when there was no evidence of full-thickness penetration of the bowel wall by cancer cells; Stage B tumours when there was full-thickness penetration, with or without involvement of adjacent structures; Stage C when tumour spread to lymph nodes was identified, and Stage D when there was metastatic spread to other organs, usually the liver.

Thirty-six patients (20 males and 16 females) with no known gastrointestinal disease were also studied as controls. These control patients were admitted to the Western Infirmary for elective surgery of minor conditions such as inguinal and femoral herniae, simple breast lumps and varicose veins. Patients were excluded from the control group if they were found to have symptoms or signs suggesting gastrointestinal disease, or if they had received an antibiotic within one month of admission. All control patients had lived in the West of Scotland for the 5 years before the study and all stated that they were eating a normal diet with no medically advised or self-imposed restrictions.

Faecal samples were obtained from the first stool passed by each patient following admission to hospital. About 0·5 g of faeces was placed in a bijoux bottle containing 4·5 ml of sterile transport medium which was then stored at −20°C to await bacteriological analysis. The remainder of the faecal sample was stored in a plastic container at −20°C to await biochemical analysis.

*Biochemical methods.*—The method used for extracting bile acids from the faeces was based on the technique first described by Evrard & Janssen (1968) and modified by Hill & Aries (1971). The deep-frozen faecal samples were weighed, homogenized with a known amount of water, and freeze dried. Steroids were extracted with glacial acetic acid and toluene. The neutral steroids were then removed using petroleum ether, and the bile acids extracted with chloroform. Sodium borohydride conversion was then carried out before re-extraction of the bile acids with ethyl acetate. Total faecal bile-acid (FBA) was then estimated using the hydroxysteroid dehydrogenase enzyme assay described by Iwata & Yamasaki (1964).

Individual bile-acid analysis (lithocholic deoxycholic and cholic acids) was carried out using aliquots of total FBA which were methylated, oxidized with chromic acid and extracted with diethyl ether. Samples were re-dissolved in acetone and aliquots injected for gas–liquid chromatography using a Pye Unicam instrument (Series 104) with an O.V.11 column. The detector was set at 275°C and the column was maintained at 175–180°C during injection of the samples. The temperature was then rapidly programmed (10°C per min) up to 250°C for chromatography of the bile-acid derivatives.

*Bacteriological methods.*—*Clostridium paraputrificum* was isolated by plating out a faecal suspension on egg-yolk agar and incubating anaerobically in Robertson's cooked meat medium. The purity of the isolates was checked by inoculation on to brain heart infusion agar plates and aerobic contaminants were identified by inoculation on to nutrient agar plates incubated aerobically. The ability of CPP to metabolize steroids was tested by incubating a culture in Todd Hewitt broth containing the substrate 5$\beta$ androstan-3, 17-dione. The presence of the unsaturated product $\Delta$4 androstan-3,17-dione, estimated by thin-layer chromatography in chloroform and acetone, indicated a culture of biochemically active CPP (NDC).

### RESULTS

The 37 colorectal cancer patients reported in this study are considered to be representative of patients presenting for treatment in the West of Scotland with this disease. The mean age and sex distribution of the colorectal cancer group (Table I) are comparable with those of

TABLE I.—*The number, age and sex distribution of the patients studied*

| Patients | No. | Male | Female | Mean age (yrs) |
|---|---|---|---|---|
| Colonic Ca | 16 | 9 | 7 | 68 |
| Rectal Ca | 21 | 12 | 9 | 65 |
| Controls | 36 | 20 | 16 | 65 |

colorectal-cancer patients in similar studies in England (Hill *et al.*, 1975) and the U.S.A. (Reddy & Wynder, 1977). The incidence of chronic irregular bowel habit (< 1 stool per 3 days or regular laxative use) and a family history of any type of cancer in the patients with colorectal cancer is shown in Table II. Two patients had been resident outside the U.K. for over 3 months but both had been living in

TABLE II.—*Clinical details of the colorectal-cancer patients*

| | Colonic cancer R n = 8 | Colonic cancer L n = 8 | Rectal cancer n = 21 |
|---|---|---|---|
| Chronic irregular bowel habit | 1 | 3 | 8 |
| Family history of cancer | 2 | 3 | 8 |
| Resident outside U.K. | 1 | 0 | 1 |

R = proximal to mid transverse colon.
L = distal to mid transverse colon.

TABLE III.—*Staging of the colorectal-cancer patients (see text for classification)*

| Stage | Colonic cancer R n = 8 | Colonic cancer L n = 8 | Rectal cancer n = 21 |
|---|---|---|---|
| A | 0 | 0 | 1 |
| B | 2 | 3 | 7 |
| C | 3 | 3 | 9 |
| D | 3 | 2 | 4 |

Scotland for at least 5 years before the diagnosis of their colorectal cancer. Details of the staging of the 37 colorectal-cancer patients are given in Table III. A full spectrum of tumour spread was observed, indicating the representative nature of the sample population.

Total FBA ($\mu$mol freeze-dried faeces) are shown in Table IV. The patients with colorectal cancer were found to have significantly less FBA than the control patients ($P < 0{\cdot}001$). Patients with colonic cancer and with rectal cancer were found to have similar total FBA concentrations. Analysis based on staging of the colorectal cancer patients as defined above revealed no statistically significant differences in total FBA concentration. Males with colorectal cancer excreted more than did females with the disease (males 14·3,

TABLE IV.—*Mean total faecal bile acid (FBA) levels and frequency (mean ± s.e.) of isolation of* Clostridium paraputrificum *(CPP) and NDC*

| | Colon n = 16 | Rectum n = 21 | Colo-rectal n = 37 | Controls n = 36 |
|---|---|---|---|---|
| FBA ($\mu$mol/g) | 13·5 ± 1·3 | 10·6 ± 0·8 | 11·8 ± 0·7 | 20·5 ± 2·2* |
| % patients with CPP | 78 | 71 | 74 | 31* |
| % patients with NDC | 72 | 57 | 64 | 15* |

* $P < 0{\cdot}001$.

TABLE V.—*Individual faecal bile acids: Mean percentage composition of the faeces from colorectal-cancer patients and controls*

| | Lithocholic acid | Deoxycholic acid | Cholic acid |
|---|---|---|---|
| Colorectal % n = 20 | $47.5 \pm 4.6$ | $48.4 \pm 3.7$ | $4.1 \pm 2.6$ |
| Controls % n = 18 | $42.8 \pm 2.8$ | $57.2 \pm 2.9$ | 0 |

females 8·4, $P < 0.001$). In the control group, however, no statistically significant difference was noted between the FBA output of males and females.

Analyses of the individual bile acids (lithocholic, deoxycholic and cholic acids) carried out on faeces from 20 colorectal-cancer patients and 18 control subjects are shown in Table V. No statistically significant difference was noted in the percentage distribution of the 3 FBAs.

Table IV gives details of the percentage of patients from whom the bacterium *Clostridium paraputrificum* (CPP) was isolated. 74% of the colorectal-cancer patients had CPP in their faeces compared with 31% of control patients ($P < 0.001$). 64% of the colorectal-cancer patients had NDC in their faeces compared with 15% of control subjects ($P < 0.001$). Analysis based on staging of the colorectal-cancer patients showed no statistically significant difference in percentage NDC isolation. Eight patients with non-malignant colonic disease have also been studied and were found to have a low (25%) incidence of NDC isolated from their faeces.

### DISCUSSION

Hill's finding of significantly increased total FBA concentrations in patients presenting with colorectal cancer has been confirmed by Reddy & Wynder (1977). These authors reported a significant rise in total FBA when they studied American Caucasians with colonic cancer and adenomatous polyps. No bacteriological evaluation was undertaken in that study, which remains the only published clinical work supporting Hill's hypothesis.

In 1977 the Intestinal Microecology Group of the International Agency for Research on Cancer reported a detailed study of sample populations from 2 areas of Denmark and Finland with a well-established 4-fold variation in the incidence of colorectal cancer. Total FBA concentrations were found to be similar in the 2 populations, and no relationship was established between cancer incidence and carriage rates of nuclear dehydrogenating clostridia (NDC). Meat consumption was greater in the high-incidence area, whereas higher intakes of dietary fibre and milk were noted in the low-incidence area.

Studies from Northern Ireland, an area with a high incidence of colorectal cancer, have failed to demonstrate an increased concentration of total FBA in either patients with colorectal cancer or patients at risk of developing this disease (colorectal adenomas, ulcerative colitis and resected colorectal tumours; Mudd *et al.*, 1978, 1979). Bacteriological analysis was not undertaken in these studies. Clearly the whole question of the association of colorectal cancer with altered faecal bile-salt metabolism demands further study.

In this study mean total FBA was found to be significantly reduced in the colorectal-cancer patients, an observation contrary to that of Hill's group. Samples have been exchanged between laboratories, and both groups are satisfied that the difference observed cannot be explained by experimental error alone. The explanation for the significant reduction in mean total FBA in our colorectal-cancer group is probably complex, but may involve increased breakdown of the bile-acid steroid molecule in the colon by bacteria. It is clear however that total FBA was not raised in our colorectal-cancer patients, and there was no evidence that one of the main individual FBAs was excreted in greater amounts at the expense of the other.

The mean total FBA of the control group of 36 patients with no known gastrointestinal disease was 20·4 μmol/g faeces. In Hill's study the corresponding value for 28 control patients without gastrointestinal disease was 13·3 μmol/g (Hill *et al.*, 1975). The 2 control groups may not be comparable, however, since Hill does not report the country of origin or dietary habits of his control patients. These may be important factors, since it is known that diet can significantly influence both colonic bile-acid concentration and the faecal flora. Hill noted a 21% incidence of abdominal pain, an 18% incidence of altered bowel habit and a 14% incidence of bleeding per rectum in his non-gastrointestinal disease control group. Patients with any of these symptoms were excluded from our control series to avoid the inclusion of patients with gastrointestinal disease as yet undiagnosed. Despite these possible differences in the control groups our results may indicate that the population of the West of Scotland excrete more FBA than their counterparts in South-East England. This finding could be related to different dietary habits in these 2 areas. A mean FBA concentration of 28·1 μmol/g has been reported from 19 control patients in Northern Ireland (Mudd *et al.*, 1979). It is interesting to note that the incidence of colorectal cancer in both the West of Scotland and Northern Ireland is greater than that recorded in the South-East of England (Calman & Kemp, 1976).

The significant increase in the isolation of NDC from faeces of colorectal-cancer patients supports the findings of Hill's group. As a result of the presence of NDC, steroid metabolism in the faeces of these patients might be enhanced, possibly leading to the formation of carcinogens or co-carcinogens as yet unidentified. This hypothesis could also explain the reduction in total FBA in patients with colorectal cancer. It is of course possible that the increased NDC isolation was secondary to the presence of the colorectal cancer, and not related in any way to its induction.

We have shown that control patients in the West of Scotland excrete considerable amounts of bile acid in their faeces and that this excretion is significantly reduced in patients with colorectal cancer. The presence of large amounts of bile acid in the colon may greatly increase the importance of the biochemically active bacteria in the colonic flora. One of these bacteria (NDC) has been isolated more frequently from the faeces of patients with colorectal cancer than from the faeces of control patients. Increased bacterial degradation of the colonic bile acids should result in less measureable bile acid in the faeces. This has been found in our study, but the nature of the degradation products remains unidentified.

Although bile-acid degradation may produce carcinogenic substances *in vitro*, a carcinogen has yet to be isolated from human faeces (La Mont & O'Gorman, 1978). The measurement of the common FBAs alone may not prove to be of value in detecting patients at risk of colorectal cancer. The search for carcinogenic degradation products in the faeces must continue, in the hope of isolating a more specific marker which might be incriminated in the aetiology of colorectal cancer.

This study was supported by a grant from the Cancer Research Campaign.

### REFERENCES

ANTONIS, A. & BERSOHN, I. (1962) The influence of diet on faecal lipids in South African white and Bantu prisoners. *Am. J. Nutr.*, **11**, 142.

ARIES, V. C., CROWTHER, J. S., DRASAR, B. S., HILL, M. J. & WILLIAMS, R. E. O. (1969) Bacteria and aetiology of cancer of the large bowel. *Gut*, **10**, 334.

BUELL, P. & DUNN, J. E. (1965) Cancer mortality among Japanese Issei and Nisei of California. *Cancer*, **18**, 656.

BURKITT, D. P. (1971) Epidemiology of cancer of the colon and rectum. *Cancer*, **28**, 3.

CALMAN, K. C. & KEMP, I. W. (1976) Gastric and colonic cancer in Scotland: Statistical review, problems and prospects in management. *Health Bull.*, **34**, 347.

DAVIS, J. A. P., KNOWELDEN, J. & WILSON, B. A. (1965) Incidence rates of cancer in Kyadondo County, Uganda 1954–60. *J. Natl Cancer Inst.*, **35**, 789.

DOLL, R. (1967) Worldwide distribution of gastrointestinal cancer. *Natl Cancer Inst. Monogr.*, **25**, 173.

DOLL, R. (1969) The geographic distribution of cancer. *Br. J. Cancer*, **23**, 1.

DOLL, R., MUIR, P. & WATERHOUSE, J. (Eds) (1970). *Cancer incidence in Five Continents*. Vol. II: Berlin:

EVRARD, E. & JANSSEN, S. (1968) Gas-liquid chromatographic determination of human faecal bile acid. *J. Lipid Res.*, **9**, 226.

GREGOR, O., TOMAN, R. & PRUSOVA, F. (1969) Gastrointestinal cancer and nutrition. *Gut*, **10**, 1031.

HAENZEL, W., CORREA, P. & CUELLO, C. (1975) Social class differences in large bowel cancer in Cali, Columbia. *J. Natl Cancer Inst.*, **54**, 1031.

HILL, M. J. & ARIES, V. C. (1971) The effect of some factors on faecal concentration of acid steroids, neutral steroids and urobilins. *J. Pathol.*, **104**, 239.

HILL, M. J., DRASAR, B. S., ARIES, V. C., CROWTHER, J. S., HAWKESWORTH, G. & WILLIAMS, R. E. O. (1971) Bacteria and aetiology of cancer of the large bowel. *Lancet*, i, 95.

HILL, M. J., DRASAR, B. S., WILLIAMS, R. E. O. & 4 others (1975) Faecal bile acids and Clostridia in patients with cancer of the large bowel. *Lancet*, i, 535.

INTERNATIONAL AGENCY FOR RESEARCH ON CANCER REPORT (1977) *Lancet*, ii, 207.

IWATA, T. & YAMASAKI, K. (1964) Enzymatic determination and thin layer chromatography of bile acids in blood. *J. Biochem.*, **56**, 424.

LA MONT, J. T. & O'GORMAN, T. A. (1978) Experimental colon cancer. *Gastroenterology*, **75**, 1157.

MUDD, D. G., MCKELVEY, S. T. D. & ELMORE, D. T. (1978) Faecal bile acid concentrations in patients at increased risk of large bowel cancer. *Br. J. Surg.*, **65**, 357.

MUDD, D. G., MCKELVEY, S. T. D., NORWOOD, W. & ELMORE, D. T. (1979) Carcinoma of the large bowel and faecal bile acids. *Br. J. Surg.*, **65**, 355.

REDDY, B. S. & WYNDER, E. L. (1977) Metabolic epidemiology of colon cancer. Faecal bile acids and neutral sterols in colon cancer patients and patients with adenomatous polyps. *Cancer*, **39**, 2533.

WYNDER, E. L. & SHIGEMATSU, T. (1967) Environmental factors of cancer of the colon and rectum. *Cancer*, **20**, 1520.

WYNDER, E. L., KAJITANI, T., ISHIKAWA, S., DODO, H., TAKANO, A. (1969) Environmental factors of cancer of the colon and rectum. II: Japanese epidemiological data. *Cancer*, **23**, 1210.